# Biorenewable FDCA-Based Alkyd Resins for More Sustainable Wood Coatings

**DOI:** 10.3390/polym17223022

**Published:** 2025-11-14

**Authors:** Victor Klushin, Ivan Zubkov, Dmitry Petrenko, Alina Petrenko, Tatyana Yurieva, Tatyana Belichenko, Aleksey Yatsenko, Yash Kataria, Anna Ulyankina

**Affiliations:** 1 Research Institute “Nanotechnologies and New Materials”, Platov South-Russian State Polytechnic University, 346428 Novocherkassk, Russia; victorxtf@yandex.ru (V.K.); ivan.n.zubkov@yandex.ru (I.Z.); dmitrypetrenko1998@yandex.ru (D.P.); alinazelens@yandex.ru (A.P.); yurevatanya06@gmail.com (T.Y.); tanya.bond.12@yandex.ru (T.B.); alexyats-npi@yandex.ru (A.Y.); katariayash1603@gmail.com (Y.K.); 2 Scientific and Educational Center “Materials”, Don State Technical University, 344002 Rostov-on-Don, Russia; 3 Resource Center for Collective Use of the Scientific and Educational Center “Materials”, Don State Technical University, 344002 Rostov-on-Don, Russia

**Keywords:** alkyd resin, 2,5-Furandicarboxylic acid, renewable resources, sustainable coating, decorative properties

## Abstract

Alkyd resins (ARs) represent a significant development in synthetic polymers, being among the oldest ones and playing a crucial role in numerous applications, especially within the coating sector. The trend is moving towards replacing non-renewable resources in the production of ARs with bio-based alternatives, with the goal of creating more sustainable binder materials as part of the transition to a bioeconomy. 2,5-Furandicarboxylic acid (FDCA) serves as a promising biomass-derived “building block” to replace non-renewable petroleum-derived aromatic diacids and anhydrides in AR synthesis. Various vegetable oils, including sunflower seed (SFO) and linseed oils (LSO), were utilized along with pentaerythritol (P) and glycerol (G) as polyols. FTIR and ^1^H NMR spectroscopies were conducted for the verification of alkyd structures. The synthesized ARs were assessed for their physico-chemical properties, including acid value, hydroxyl value, color, density, and viscosity. The performance of the resulting alkyd coatings, which are crucial for their commercial applications, was examined. Key factors such as drying time, hardness, adhesion, wettability, chemical and corrosion resistance, and UV stability were analyzed. All synthesized FDCA-based alkyd coatings demonstrate outstanding adhesion, good thermal stability up to 220 °C, and barrier properties for steel with |Z|_0.02Hz_ ~10^6^–10^7^ Ohm cm^−2^, which render them suitable for the processing requirements of indoor coating applications. The higher temperature at 50% mass loss (T_50_) for SFO-P (397 °C) and LSO-P (413 °C) as compared to SFO-G (380 °C) and LSO-G (394 °C) indicated greater resistance to thermal breakdown when pentaerythritol was used as a polyol. Replacing glycerol with pentaerythritol in FDCA-based ARs resulted in a viscosity increase of 1.2–2.4 times and an enhancement in hardness from 2H to 3H. FDCA-based ARs exhibited decreased tack-free time, enhanced thermomechanical properties, and similar hardness as compared to phthalic anhydride-based ARs, underscoring the potential of FDCA as a sustainable alternative to phthalic anhydride in the formulation of ARs, integrating a greater proportion of renewable components for wood coating applications.

## 1. Introduction

Alkyd resins (ARs) represent a significant category of synthetic polymers, with a long-standing history and extensive use in numerous applications, especially within the coating industry. They serve as binders in paint and varnish formulations, accounting for a large volume of coatings used for decorative purposes [1]. The compatibility of ARs with various polymers and the broad formulation options make them suitable for creating a wide range of coating materials, including products like do-it-yourself paints and varnishes for wood and metal, road marking paints, metal anticorrosive primers, architectural coatings, acid curing coatings, and others [2,3,4,5].

ARs generally consist of as much as 80% bio-based components, especially fatty acids and polyols, which can be readily sourced from biofuel production and agro-industrial waste. Glycerol and pentaerythritol are among the most commonly utilized polyols for the production of ARs [6]. Nonetheless, the diacid building blocks continued to be sourced from petrochemical resources. In response to environmental pressure and depletion of fossil fuels, manufacturers of ARs initiated the development of a more environmentally friendly version of their products [2]. The trend is moving towards replacing non-renewable resources with bio-based alternatives, aiming to develop more sustainable polymer materials as part of the transition to a bioeconomy [3,7,8]. 2,5-Furandicarboxylic acid (FDCA) serves as a biomass-derived green replacement for non-renewable monomers, including aromatic diacids and anhydrides such as phthalic compounds, in the synthesis of polyethylene 2,5-furandicarboxylate [9], as well as FDCA-based polyesters [10], polyurethanes [11] and polyamides [12]. The adaptability of FDCA in producing high-performance polymers, along with the increasing regulatory support for sustainable materials and the movement towards a circular economy, is fostering the adoption of FDCA across various sectors [13]. Furthermore, FDCA is known for its unique stable structure, preventing polymer chains from moving and deforming easily under stress. The rigidity of its five-membered ring significantly improves the thermomechanical and barrier characteristics of polymers, setting them apart from other long-chain bio-monomers. The structure, combined with the polarity imparted by the oxygen atom, indicates that the furan ring presents greater resistance to flipping compared to phthalic acid derivatives [14].

Although the potential is obvious, FDCA has not garnered significant focus regarding its use in ARs. Jan Janesch and co-workers have proposed application of furan-2,5-dicarboxylic acid dimethyl ester (FDME), a derivative of FDCA, in ARs [15]. Nonetheless, there was a scarcity of data regarding the functional properties of the resulting polymers when used as coating materials.

The composition of ARs, particularly the type and ratio of their components such as oil, polyol, and dibasic acid, can influence their properties and performance, including factors like drying time, flexibility, hardness, durability, and other characteristics [2]. Linseed oil, followed by soybean oil, rapeseed oil, castor oil, and sunflower oil are primarily used in the large-scale production of ARs [6]. Linseed and sunflower oils exhibit similar chemical structures, yet they possess distinct chemical compositions. Linseed oil is composed of the triply unsaturated α-linolenic acid (49.85%) and the doubly unsaturated linoleic acid (16.67%), demonstrating a greater level of unsaturation compared to sunflower oil [3].

In light of the demand for more sustainable coating materials and the insufficient exploration of the potential of FDCA as a replacement of phthalic anhydride in AR production, this study focused on the synthesis and extensive characterization of FDCA-based ARs. The effect of various vegetable oil fatty acids, specifically sunflower seed and linseed oils, along with pentaerythritol and glycerol as polyols, on FDCA-based AR properties was investigated. The potential of FDCA in developing more sustainable wood coatings that incorporate a higher proportion of renewable components has been emphasized.

## 2. Materials and Methods

The synthesis of FDCA was carried out following the method outlined in the article [16]. Unrefined sunflower oil, linseed oil, pentaerythritol, and ortho-xylene were acquired from LLC GK “Ruskhim”, Ekaterinburg, Russia. Glycerin was purchased from JSC “KupavnaReaktiv”, Stary Kupavna, Russia. Sodium carbonate decahydrate was purchased from OJSC “Mikhailovsky Chemical Reagents Plant”, Barnaul, Russia. Cobalt octoate CO-1 (12% cobalt) and calcium octoate CO-4 (5% calcium) were kindly supplied by LLC “Himpostavshchik Don”, Aksai, Russia. Maleic anhydride and zinc acetate were purchased from LLC “JSC REAKHIM”, Moscow, Russia. Hexane was purchased from LLC “KHIMPROM-M”, Yaroslavl, Russia. Heptane was obtained from LLC “NPP Aquatest”, Rostov-on-Don, Russia. Isopropyl alcohol, acetone, toluene, and benzene were purchased from JSC “Ecos-1”, Moscow, Russia. Sodium chloride was purchased from JCS “LenReactive”, Saint Petersburg, Russia. Commercial alkyd resin (CAR) (pentaphthalic varnish, PP-231) was purchased from GoodMaster Ltd., Saint Petersburg, Russia.

**Figure 1:**
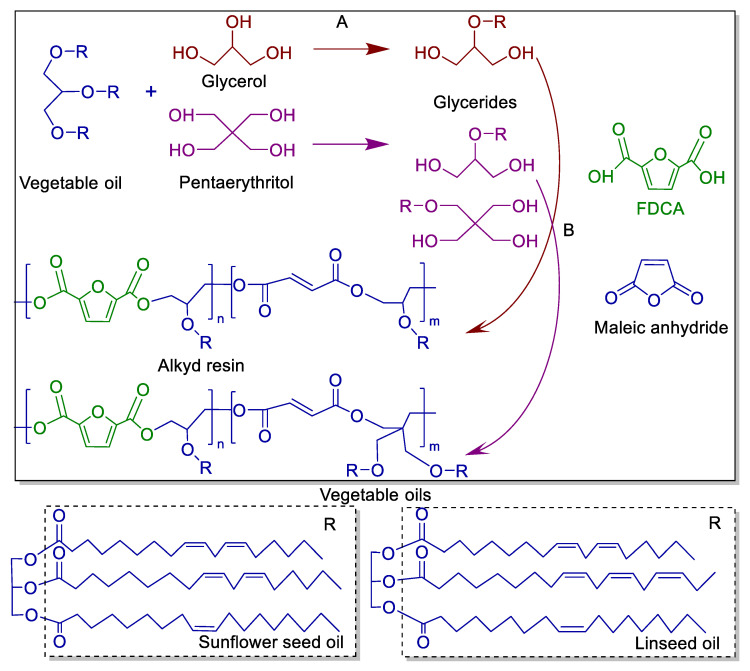
The synthetic pathway of FDCA-based ARs. A: monoglyceride process; B: polycondensation process.

Two-step synthesis was used to obtain FDCA-based ARs. Alcoholysis, or monoglyceride process, is the initial step in converting sunflower seed oil or linseed oil into monoglycerides (step A). The second step of the esterification procedure involves adding FDCA, maleic anhydride and xylene to the mixture (step B). Figure 1 depicted the FDCA-based AR synthesis route. The resin formulations are summarized in Table 1. Parts by weight were used to specify the relative proportions of ingredients by mass, allowing for scaling recipes up or down by multiplying the parts by any convenient weight unit.

Sunflower-seed/linseed oil (14.0 g), glycerol (1.76 g) or pentaerythritol (1.95 g), along with Na_2_CO_3_ (0.56 g) were charged into a 0.25 L three-necked round-bottom flask equipped with a mechanical stirrer (IKA microstar 7.5 control) and a temperature controller. The setup for the synthesis was illustrated in Figure S1. The reaction mixture was heated to 220 °C for 1.5 h and stirred 250 rpm until monoglyceride was formed. Monoglyceride formation was confirmed by a 95% ethanol tolerance test. One part of the reaction mixture was mixed with 5 parts of ethanol. A completely clear solution of reaction mixture in 95% ethanol indicated the formation of the monoglyceride [17]. Then, the reaction mixture was cooled to 150 °C, and Dean–Stark trap and a reflux condenser were connected to the flask. In the second stage, the polycondensation reaction was carried out by adding maleic anhydride (0.2 g), FDCA (3.98 g) or PA (3.78 g), and zinc acetate (0.02 g). Water was removed as a by-product by azeotropic distillation using xylene (5 mL) as a solvent. The reaction mixture was further heated to 240 ± 5 °C. The reaction was quenched by allowing it to cool when the acid value was less than 20 mg KOH per g. The alkyd resins were diluted to ~60% of solid content (non-volatile matter content) by adding o-xylene as an acceptable solvent for green production of alkyd resins [18]. Non-volatile matter content was determined according to ISO 3251:2019 [19]. A mixture of cobalt and calcium salts was used as a drying catalyst in all experiments. The resulting mixtures were stirred to give homogeneous solutions and then stored overnight prior to their application on test plates. AR samples were labeled by the vegetable oil and polyol used during synthesis. SFO and LSO labels were used for sunflower seed and linseed oils, respectively. G and P labels were used for glycerol and pentaerythritol, respectively. Phthalic anhydride-based AR using glycerol and sunflower oil (PA-based SFO-G) was synthesized to compare its performance with the performance of FDCA-based ARs. The formulation is presented in Table 1.

Fourier-transform infrared (FTIR) spectra of the specimens were recorded on a WQF-530 spectrometer (BFRL, China) over a 4000–400 cm^−1^ frequency range using KBr as a reference. The molecular structure of the ARs was determined from ^1^H NMR measurements, which were performed on an Avance 300 MHz (Bruker, Rheinstetten, Germany) spectrometer. Samples were dissolved in deuterated CDCl_3_. Thermogravimetric measurements (TG) were performed using STA 449 F5 (Netzsch, Selb, Germany) under helium atmosphere (70 mL min^−1^) at heating rate 10 K min^−1^. The heating rate varied between 5, 10, 15, and 20 K min^−1^ to estimate the activation energy (*E_a_*).

The acid value (AV) of the ARs was determined by a volumetric titration with 0.1 M KOH in EtOH using phenolphthalein as a color indicator according to ISO 660:2020 [20]. The hydroxyl value (HV) was determined by the esterification of the hydroxyl groups using a phthalic anhydride solution in pyridine with a final titration of the excess acid reagent by a previously standardized sodium hydroxide solution according to ISO 4327-1979 [21]. The density of the ARs was calculated from the mass of the product in the pycnometer and the known volume of the pycnometer according to ISO 2811-1:2023 [22]. Viscosity was determined in accordance with the ASTM (D445-24) [23] by measuring the time for a volume of liquid to flow under gravity through a calibrated glass capillary viscometer (capillary diameter of 1.77 mm) at a temperature of 25 ± 0.5 °C. The Gardner color scale was used to compare the sample with the liquid standards, which are a series of 18 color variations ranging from near clear, light yellow (Gardner 1) to dark brown (Gardner 18).

The films were cast on the glass (1.5 × 25 × 75 mm) for evaluating UV resistance and wettability (contact angle) or metal (3 × 20 × 50 mm) panels for evaluating drying time, shear strength, hardness, adhesion, and corrosion resistance with a 60 μm applicator bar. The coatings were left to dry for 72 h at an ambient temperature prior to measurements. The surface of metal plates was cleaned from rust, grease, and other oxides using the mixture of hydrochloric acid (69%), water (26%), and urotropine (5%), sanded with sandpaper (P1500), degreased with acetone, and dried at the ambient conditions. The glass plates were degreased with acetone. The film thickness was measured using 0 to 25 mm a micrometer, and the average thickness obtained was 50 (±4) μm, which was controlled to allow a comparative analysis of the different coatings. Determination of the time and degree of drying of alkyd coatings was in accordance with ISO 9117-4:2012 [24]. The shear strength was determined using a shear test in a REM 20 universal testing machine (Metrotest, Russia). The surface treatment of steel plates for bonding involved coating the adherend surfaces with the synthesized AR. Epoxy adhesive was used to produce the adhesive joints [25]. A crosshead speed of 5 mm/min and a loading of 0–20 kN were used. Hardness was determined using a pencil test according to ISO 15184:2020 [26]. The adhesion of the coatings to the substrate was characterized using the cross-cut test, which is a simple and effective method for evaluating adhesion involving a cut of a grid pattern into the coating and assessing the adhesion according to ISO 2409:2020 [27]. The resistance of the coatings to the solvents (n-hexane, n-heptane, isopropanol, acetone, toluene, and benzene) was determined in accordance with ISO 2812-4:2017 [28]. The test substances were applied to the test panels with a pipette for 1 h, and after that, wiped with dry cotton wool. The contact angle of the paint film was measured using a microscope. A 2 μL water droplet was placed on the surface of the samples, and the contact angle was recorded after a 5 s waiting period. The coated carbon steel samples were investigated by means of electrochemical impedance spectroscopy (EIS), employing a SmartStat (Electrochemical Instrument, Russia). A perturbation amplitude of 20 mV was applied at the open circuit potential (OCP) to collect EIS spectra over the frequency range 10^4^–10^−2^ Hz in 3.5 M NaCl solution. The exposed surface area of the samples was 1 cm^2^. All measurements were conducted by using a typical three-electrode cell where an Ag/AgCl electrode (3.5 M KCl) was used as a reference electrode with a platinum wire as a counter electrode. The data were fitted by using the program Zview. To examine the UV resistance of the alkyd coatings, an accelerated degradation test was conducted using a homemade UV weathering test chamber equipped with a UVA-351 lamp according to ISO 16474-3:2021 [29]. The spectral irradiance at 340 nm was 0.76 W m^−2^ nm^−1^. The temperature of the alkyd coatings was approximately 50 °C. The irradiation time was 24 h. The salt spray test was used to check the corrosion resistance of the coatings using a salt spray chamber (chamber capacity of 240 L, temperature of 40 °C, salt spray fall-out rate of 4.0 mL h^−1^, pH of 7). The samples were placed in the chamber and exposed to salt mist for 3 h and humidity for 1 h. After the experiment, samples were rinsed and dried and then they were observed whether changes occurred on their surface.

## 3. Results and Discussion

### 3.1. Structural Characterization of FDCA-Based ARs

**Figure 2:**
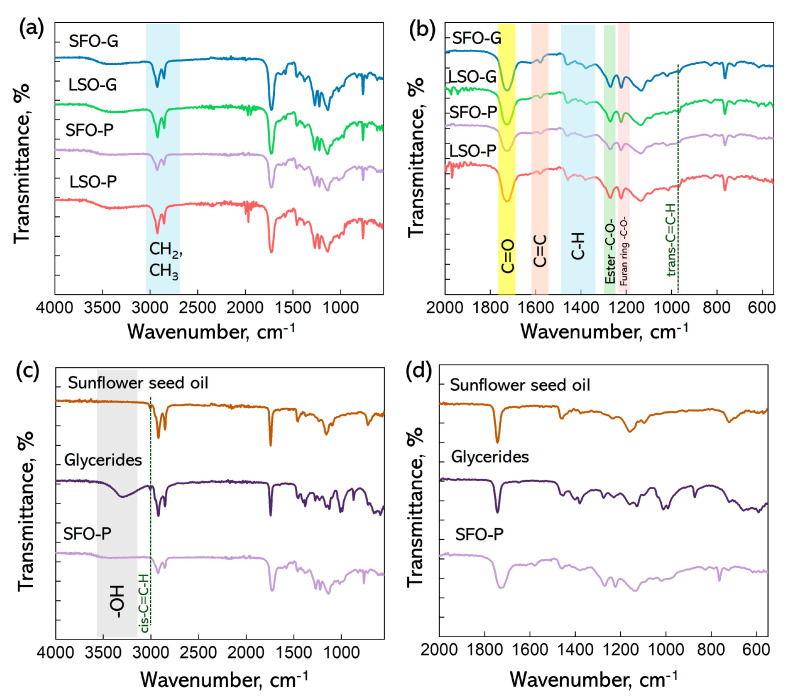
FTIR spectra (**a**,**c**) and enlarged FTIR spectra in the region of 2000–500 (**b**,**d**) of the FDCA-based ARs.

FTIR spectroscopy was employed to examine the bonding structure of the synthesized polymers. The FTIR spectra of all alkyd resins (Figure 2a) were found to be similar to each other. Only small intensity redistribution between some spectral bands was noted. The most intense signal at 1726 cm^−1^ (Figure 2b) corresponds to the stretching vibration of the ester carbonyl group C=O [15,30]. Two signals at 2925 and 2860 cm^−1^ may be ascribed to the C–H stretching vibrations of –CH_2_ and –CH_3_ groups, respectively, while the bending C–H vibrations of –CH_2_ and –CH_3_ can be found at 1461 and 1384 cm^−1^ [30]. The peaks at 1270 and 1137 cm^−1^ are characteristics of an ester group. Peaks in the alkyd samples, which stem from the presence of FDCA and its reaction products, along with maleic anhydride, are found at a wavenumber of 1583 cm^−1^ related to the -C=C- of the furan ring, 1223 cm^−1^ from the C-O group of the furan ring, and 1460 and 1385 (–CH– deformations and wagging peaks), 1221 and 1016 (=C–O–C= stretching vibrations in the furan ring), 966 and 765 (C–H bending vibrations in the furan ring) [15,31]. Intensification of an absorption band at 967 cm^−1^ was observed for the LSO-modified coatings. This band is assigned as the *trans* double bonds in fatty acids. LSO is mainly composed of linolenic acid, which is more susceptible to oxidation than linoleic acid as the main fatty acid of SFO. One of the results of oxidation is the conversion of *cis* isomers to *trans* ones [32].

The FTIR method can be used to control the stages of the monoglyceride process and polyesterification [33]. In vegetable oil, the -O-H stretching vibration in the region of 3000–3500 cm^−1^ is absent due to the lack of free hydroxyl groups. The glyceride sample shows a broad prominent band due to the introduction of hydroxyl groups as a result of the reaction with pentaerythritol (Figure 2c). The disappearance of the O-H band at ~3400 cm^−1^ in the FTIR spectrum of the product after the polycondensation stage provides another argument in favor of the successful esterification reaction between glycerides, FDCA, and maleic anhydride [15,33]. The symmetrical CH stretching band of the *cis*-CH=CH moiety in vegetable oil and glycerides can be observed at 3008 cm^−1^. The disappearance of this band in the cured AR can be indicative of the autoxidation process [34].

The C=O stretching band has a lower wavenumber of around 1726 cm^−1^ in alkyd samples compared to the vegetable oil and glycerides sample (1742 cm^−1^). The observed downshift in the alkyd indicates a modification in the electronic environment surrounding the carbonyl group. The electron-donating effect of the neighboring aromatic group increases the C-O bond length in the carbonyl group, confirming the presence of conjugation within the alkyd structure [15].

**Figure 3:**
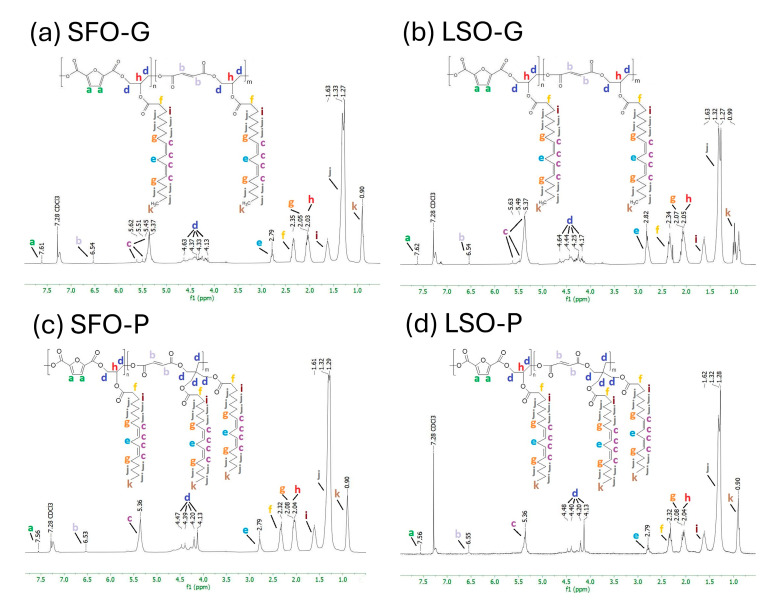
^1^H NMR spectra of FDCA-based ARs.

Figure 3a–d shows the ^1^H NMR spectra that contain the signals typical of an AR classified with letters starting from the low-field region. The potential positions of protons associated with each peak in the structures of alkyd resins, which represent possible repeating units, are identified in Figure 3 for each sample. The signal observed at *δ* 7.28 ppm is attributed to chloroform-d. Through the examination of the spectra acquired for the sunflower seed oil and the glycerides (Figure S2), the presence of fatty acid protons and polyol protons was noted, while signals associated with aromatic protons were absent. The synthesized AR shows that the low-field region of the spectrum at *δ* = 7.56–7.62 ppm (peak a) corresponds to the protons of the furan ring. The resonance observed in the range of *δ* = 6.53–6.55 ppm (peak b) can be attributed to the presence of maleic ester moieties. The resonance observed in the range of *δ* = 5.37–5.62 ppm, identified as peak c, was attributed to the vinylic protons. The signals *δ* = 4.13–4.64 ppm, consisting of peaks d, are attributed to the protons associated with the CH_2_ groups of the polyol(s). The resonance observed at approximately *δ* = 2.79–2.82 ppm (peak e) has been attributed to aliphatic CH_2_ groups that are shielded by two adjacent vinyl groups. The peaks that persist in the aliphatic region *δ* = 0.5–2.6 ppm are attributed to sp^3^ C-bound protons associated with the fatty acid chains (peaks f–k). The assignment of ^1^H-NMR chemical shifts in SFO-G, LSO-G, SFO-P, and LSO-P AR is:

SFO-G: ^1^H NMR (300 MHz): 0.9 (s, 3H, CH_3_) (-C**H**_3_), 1.30 (d, J = 17.5 Hz, 10H, 5CH_2_) (-C**H**_2_-), 1.63 (s, 2H, CH2) (-C**H**_2_-CH_2_-CO-), 2.04 (d, J = 7.2 Hz, 3H, CH, CH_2_) (>C**H**-O-CO- and -C**H**_2_-CH=CH-), 2.35 (s, 2H, CH_2_) (-C**H**_2_-CO-), 2.79 (s, 2H, CH_2_) (-CH=CH-C**H**_2_-CH=CH-), 4.27 (d, J = 31.5 Hz, 2H, CH_2_) (-C**H**_2_-O-CO-), 4.54 (d, J = 58.3 Hz, 2H, CH_2_) (-C**H**_2_-O-CO-R), 5.37 (s, H, CH) (-**H**C=C**H**-), 6.54 (s, H, CH) (-CO-C**H**=C**H**-CO-), 7.61 (s, H, CH) (Ar-C**H**=C<CO-)
LSO-G: ^1^H NMR (300 MHz) 0.99 (s, 3H, CH_3_) (-C**H**_3_), 1.30 (d, J = 14.4 Hz, 10H, 5CH_2_) (-C**H**_2_-), 1.63 (s, 2H, CH_2_) (-C**H**_2_-CH_2_-CO-), 2.06 (d, J = 6.3 Hz, 3H, CH, CH_2_) (>C**H**-O-CO- and -C**H**_2_-CH=CH-), 2.34 (s, 2H, CH_2_) (-C**H**_2_-CO-), 2.82 (s, 2H, CH_2_) (-CH=CH-C**H**_2_-CH=CH-), 4.21 (d, J = 23.7 Hz, 2H, CH_2_) (-C**H**_2_-O-CO-), 4.54 (d, J = 60.5 Hz, 2H, CH_2_) (-C**H**_2_-O-CO-R), 5.37 (s, H, CH) (**-H**C=C**H**-), 6.54 (s, H, CH) (-CO-C**H**=C**H**-CO-), 7.62 (s, H, CH) (Ar-C**H**=C<CO-)
SFO-P: ^1^H NMR (300 MHz) 0.9 (s, 3H, CH_3_) (-C**H**_3_), 1.30 (d, J = 9.0 Hz, 16H, 8CH_2_) (-C**H**_2_-), 1.61 (s, 4H, 2CH_2_) (-C**H**_2_-CH_2_-CO-), 2.06 (d, J = 10.2 Hz, 6H, 2CH, 2CH_2_) (>C**H**-O-CO- and -C**H**_2_-CH=CH-), 2.32 (s, 2H, CH_2_) (-C**H**_2_-CO-), 2.79 (s, 2H, CH_2_) (-CH=CH-C**H**_2_-CH=CH-), 4.16 (d, J = 21.5 Hz, 2H, CH_2_) (-C**H**_2_-O-CO-), 4.43 (d, J = 23.6 Hz, 2H, CH_2_) (-C**H**_2_-O-CO-R), 5.36 (s, H, CH) (**-H**C=C**H**-), 6.53 (s, H, CH) (-CO-C**H**=C**H**-CO-), 7.56 (s, H, CH) (Ar-C**H**=C<CO-)
LSO-P: ^1^H NMR (300 MHz) 0.9 (s, 3H, CH_3_) (-C**H**_3_), 1.30 (d, J = 11.8 Hz, 16H, 8CH_2_) (-C**H**_2_-), 1.62 (s, 2H, CH_2_) (-C**H**_2_-CH_2_-CO-), 2.06 (d, J = 12.5 Hz, 3H, CH, CH_2_) (>C**H**-O-CO- and -C**H**_2_-CH=CH-), 2.32 (s, 2H, CH_2_) (-C**H**_2_-CO-), 2.79 (s, 2H, CH_2_) (-CH=CH-C**H**_2_-CH=CH-), 4.17 (d, J = 21.0 Hz, 4H, 2CH_2_) (-C**H**_2_-O-CO-), 4.44 (d, J = 24.6 Hz, 2H, CH_2_) (-C**H**_2_-O-CO-R), 5.36 (s, 2H, 2CH) (**-H**C=C**H**-), 6.55 (s, H, CH) (-CO-C**H**=C**H**-CO-), 7.56 (s, H, CH) (Ar-C**H**=C<CO-)

The proton resonance assignment of all peaks aligns with the data in the literature [35]. Hence, from the ^1^H-NMR results, it can be concluded that FDCA-based ARs were successfully prepared, which is also consistent with the above FTIR results.

### 3.2. Physico-Chemical Properties of FDCA-Based ARs

The physico-chemical properties of FDCA-based ARs are exhibited in Table 2. AV and HV are vital parameters for Ars, representing chemical and corrosion resistance, adhesion and drying rate, flexibility, and storage stability. All the ARs have the AV below 15 mg KOH g^−1^ making them preferred binders for paint and varnish manufacturing [36]. HVs (below 70 mg KOH/g) were also within acceptable limits, demonstrating their suitability for industrial applications. The density of the ARs modified with sunflower seed oil was in the range of 0.746–0.758 g cm^−3^, while the density of the AR modified with linseed oil ranged from 0.855 to 0.887 g cm^−3^. Pentaerythritol imparted higher viscosity to FDCA-based ARs as compared to glycerol; that can be due to the higher degree of branches [37].

Rheological parameters are crucial for industrial coatings. A knowledge of formulation rheometry allows optimizing the production, storage, and stability of the final products, also providing important information on their application and final properties of coatings in terms of surface smoothness and homogeneity. Viscosity is a critical property in polymer processing, influencing both product quality and manufacturing efficiency [38]. Incorrect viscosity can result in defects, such as uneven textures or surfaces. The fluidity property of the solution is regulated by varying the amount of solvent in the solution. Low-viscosity varnishes (50–200 mm^2^ s^−1^) are applied to the surface by spraying or dipping, forming a thin layer with possible defects in the form of runs. High-viscosity varnishes (500–2500 mm^2^ s^−1^) are applied to surfaces with a brush or roller, resulting in a thicker layer. A possible defect in this case is the appearance of unevenness (“orange peel”). In this work, varnishes were obtained with kinematic viscosity ranging from 51.0 to 684.6 mm^2^ s^−1^. SFO-G and SFO-P samples, exhibiting a viscosity of 570.4–684.6 mm^2^ s^−1^, are preferable for application by brush or roller. LSO-G, exhibiting a viscosity of 51.0 mm^2^ s^−1^, is suitable for application to surfaces by spraying with layer thickness control—applying a large amount simultaneously can lead to defects in the form of runs. In turn, LSO-P, exhibiting a kinematic viscosity of 124.2 mm^2^ s^−1^, is optimal for most methods of applying paint and coatings. It is important to note that by adjusting the viscosity before use (adding additional solvents, preheating, etc.), the application method of the varnish can be changed without altering its performance characteristics [39].

The aesthetic appearances of synthesized ARs were investigated and summarized in Table 2, and the color variation could be attributed to the conditions of alkyd production and the nature of the fatty acid used in the AR preparation [18].

### 3.3. Coating Properties of the FDCA-Based ARs

Some of the important coating properties, which are paramount to the commercial viability and quality of ARs, including drying time, shear strength, hardness, adhesion, corrosion, and wettability, are summarized in Table 3.

Drying time is a crucial property for the application of ARs as binders in paint, as it determines the ability of AR to dry hard through autoxidation and create durable films [40]. The drying mechanism of an alkyd coating is intricate and is typically divided into two stages. The initial stage involves the solvent evaporation, followed by the oxidative drying of the fatty acid chains, ultimately leading to the formation of a polymer network. Co-based driers serve as the predominant primary catalyst in alkyd coatings and are frequently used alongside secondary driers (Ca, Zr) to improve the drying process of the film. The Co-based drier demonstrates front drying, whereas incorporating the Ca-based secondary drier improves the through-drying of the coating and strengthens the intermolecular networks [41,42]. The drying schedule for the various alkyd samples was determined by measuring the time required for the resin to harden, specifically the tack-free time. (Table 3). This parameter plays an essential role in understanding the rate and extent of drying of the coating once it is applied to a surface. A reduction in tack-free time from 5 to 3 h was observed with an increase in HV from 29.3 to 69.5. The HV, representing the concentration of free hydroxyl groups in a polymer, significantly influences oxidative polymerization. The presence of hydroxyl groups allows for reactions with radicals produced during oxidation when a dryer is present, which can influence the reaction rate, crosslinking density, and the overall structure of the polymer [43]. Furthermore, the faster tack-free time is commonly observed in linseed oil modified resins, attributed to the increased unsaturation present in the linseed oil [44]. FDCA-based ARs exhibited decreased tack-free time (5 h for SFO-G) as compared to phthalic anhydride-based AR (6 h for PA-SFO-G). While the final cross-linking (auto-oxidation) of ARs primarily occurs via the unsaturated fatty acids, the initial “tack-free” time is significantly influenced by the physical properties of the initial resin, especially its molecular weight and T_g_. Incorporation of FDCA can yield resins with higher molecular weight and increased viscosity as outlined in [15].

The shear strength of a varnish coating pertains to its ability to withstand forces exerted parallel to the coating surface, which may lead to sliding or detachment from the substrate. This property is essential for maintaining the durability and longevity of the coating, particularly in scenarios where the coated surface undergoes stress or friction. The shear strength of linseed oil-modified AR showed an increase when compared to sunflower seed oil-modified AR (Table 3). Linseed oil exhibits significant levels of unsaturation and enhances the crosslinking process and/or polymerization per unit weight of material, leading to a more robust and durable product [6,32].

The pencil hardness test is a commonly employed technique for evaluating scratch resistance [45,46]. The hardness is determined by the pencil that inflicts lasting damage to the coating. The incorporation of pentaerythritol into the formulations demonstrated an enhancement in the hardness of the coatings, increasing from 2H to 3H. The increased hardness is explained by higher functionality and, consequently, greater cross-linking density. The hardness of biorenewable FDCA-based ARs using pentaerythritol as a polyol component was found to be higher than that of the commercial AR, which exhibited a 2H hardness, as outlined in [47]. A tung oil-based acrylated-alkyd resin synthesized from tung oil, phthalic anhydride and modified with isobornyl acrylate showed pencil hardness values between H and 3H [17].

**Figure 4:**
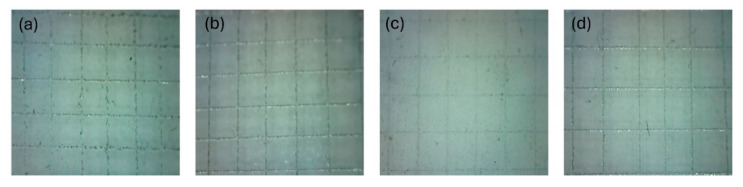
Evaluation of adhesion by cross-cut test for SFO-G (**a**), LSO-G (**b**), SFO-P (**c**), LSO-P (**d**).

Adhesion is largely dependent on surface affinity and wetting. The inclusion of polar carboxyl groups from FDCA and ester carbonyl groups along the polymer chain promotes the formation of hydrogen bonds with hydroxyl groups on the metal surface, thereby enhancing adhesion. The characterization of film adhesion to the substrate was conducted utilizing the crosshatch adhesion method. This method employed a rating system to indicate the extent of adhesion loss. The results are meticulously examined with the aid of a magnifying glass. The reproducible adhesion test results demonstrated that all synthesized ARs exhibited outstanding adhesion to metal plates, as indicated by the completely smooth cut edges (classification 0—0% detached coating) (Figure 4).

**Figure 5:**
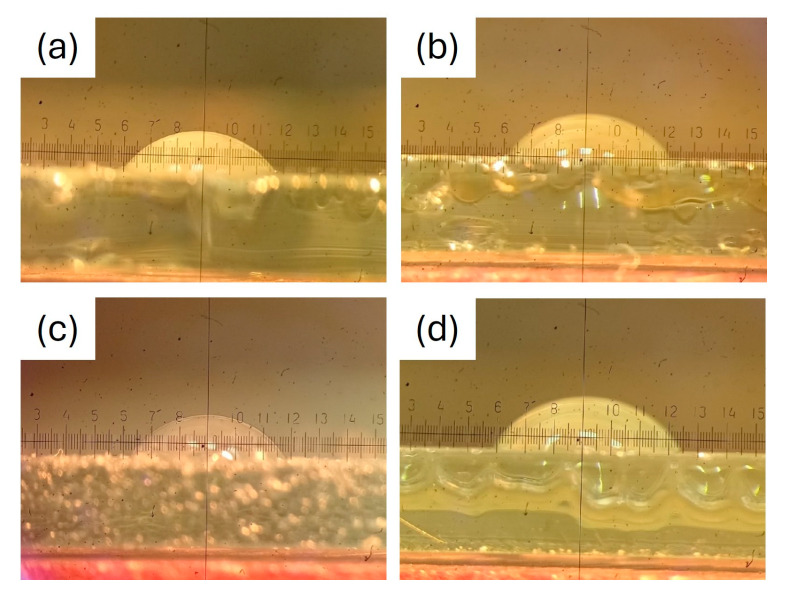
Evaluation of contact angles for SFO-G (**a**), LSO-G (**b**), SFO-P (**c**), LSO-P (**d**).

Figure 5 demonstrates the assessment of contact angles for the coatings. The values for the contact angles of the coatings are detailed in Table 3. The contact angle increases with an increase in surface hydrophobicity [2]. Smaller contact angles suggest a higher affinity of the surface for water, leading to greater spreading of water across the surface. Conversely, larger contact angles indicate unfavorable wetting properties and a reduced affinity of the film surface for water. Using the values of water contact angles, the sunflower seed oil-modified coatings can be classified as weakly hydrophilic, while the linseed oil modified coatings are weakly hydrophobic surfaces [48]. LSO-modified alkyd coatings demonstrated a higher contact angle in comparison to SFO. This could stem from the oxidative polymerization and drying of LSO, which limits the molecular mobility of the liquid oil and leads to the formation of a nearly solid surface, as reported elsewhere [32]. The confirmation of this phenomenon was achieved through FTIR analysis.

**Figure 6:**
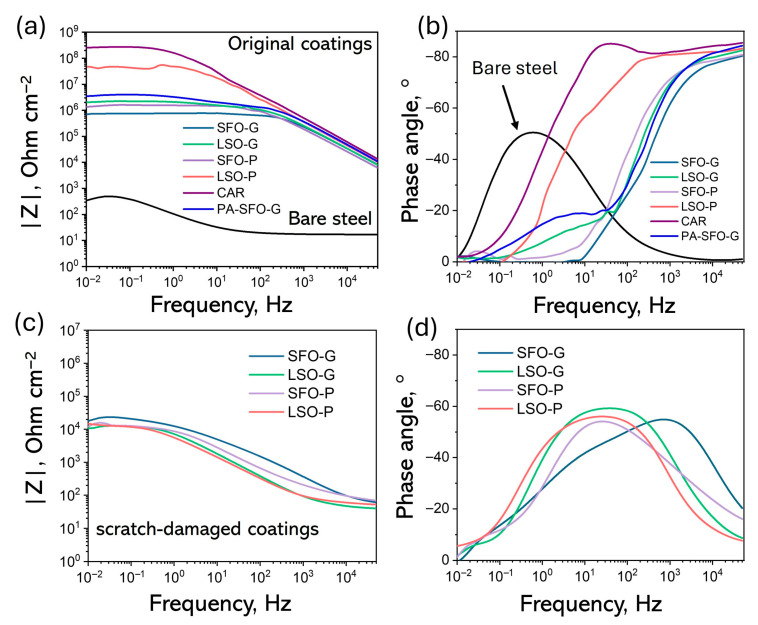
Bode-modulus (**a**,**c**) and Bode-phase plots (**b**,**d**) of the FDCA-based, CAR and PA-SFO-G alkyd coatings after 30 min of corrosion test (**a**,**b**) and after scratch-damaging (**c**,**d**).

EIS is considered to be very important as it allows for the examination of both the dielectric properties of the film and the corrosion processes occurring at the metal/film interface [49]. EIS measurements were conducted following a 30 min immersion in a 3.5% NaCl solution at ambient temperature. The Bode diagrams derived from the tests are shown in Figure 6. The film capacitance (*C_f_*) is predominant at higher frequencies, while the pore film resistance (*R_pore_*), which refers to the resistance of the solution contained within the pores and defects of the paint film, appears at intermediate and lower frequencies. The value of |Z| at the lowest frequency serves as a semi-quantitative indicator of coating barrier performance, since the low frequency range of EIS measurements aligns with the region where the electrochemical process takes place at the metal/electrolyte interface [50]. Typically, a |Z| ≥ 10^6^ Ω cm^−2^ is required for a coating to exhibit an adequate level of corrosion protection as outlined in [51]. Nonetheless, the impedance of real coating is influenced by the method of application, as this can lead to the creation of defects and variation in film thickness [52]. The comparison of the Bode plots for bare steel substrate, synthesized alkyd coatings, and CAR, all with a similar thickness of approximately 50 µm and measured under identical conditions, was conducted.

The synthesized ARs maintained the |Z|_0.02Hz_ range from ~7.3·10^5^ to 4.5·10^7^ Ω·cm^−2^. The |Z|_0.02Hz_ values for LSO-G, SFO-P and LSO-P exceed 10^6^ Ω cm^−2^, indicating effective prevention against the erosion of corrosive media to the substrate and exhibiting high corrosion resistance, comparable to that of CAR (Figure 6a). Furthermore, the phase angle approaching 90° in the high-frequency region indicates a remarkable barrier property of the coating following 30 min of immersion (Figure 6b). The Bode graphs of the scratch-damaged samples (Figure 6c,d) illustrate a notable reduction in barrier properties and indicate substrate activity at mid to low frequencies. This aligns with the double-layer capacitance observed at the steel surface, particularly in regions exposed at the base of pores and defects or where the coating has delaminated [53]. The difference in corrosion resistance between the original samples and the scratch-damaged samples is evident.

The corrosion state of the samples after the salt-spray chamber (temperature of 40 °C, salt spray fallout rate of 4.0 mL h^−1^, pH of 7, exposure to salt mist for 3 h, and humidity for 1 h) is shown in Figure S4.

The excellent resistance of the coatings to n-hexane, n-heptane, and isopropanol indicated that the solvents were not trapped in the dried film (Table 4). The lower chemical resistance of alkyd coatings to acetone, toluene, and benzene is commonly observed in the literature [18].

### 3.4. Thermal Stability of the FDCA-Based Alkyd Coatings

**Figure 7:**
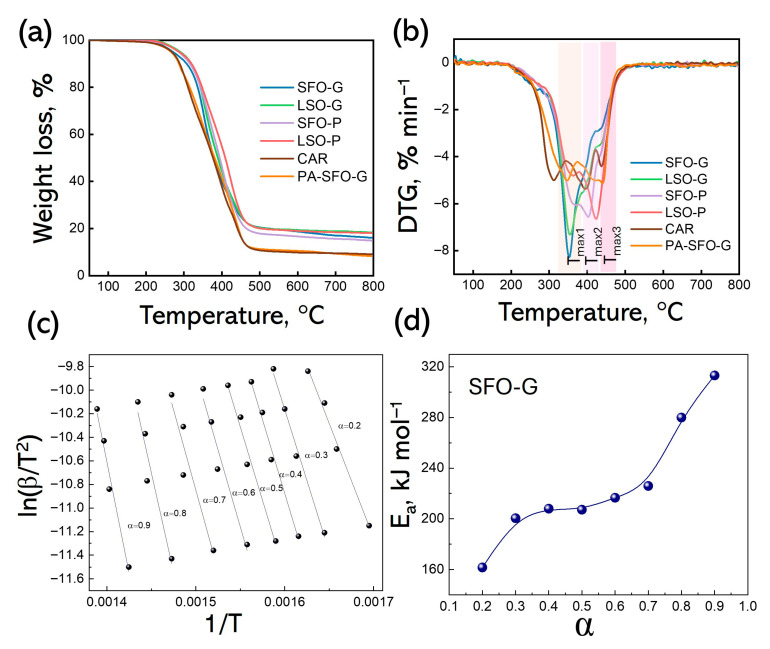
TG (**a**) and DTG (**b**) curves of FDCA-based, CAR and PA-SFO-G alkyd coatings; Fitting curves for thermodynamic equations (KAS method) (**c**) and plot of *E_a_* vs conversion rate for SFO-G (**d**).

Thermal stability is a significant factor for the properties of the coatings. The thermal behavior of the synthesized AR was studied. The synthesized resins exhibit moderate thermal stability under an inert atmosphere, with degradation occurring above 200 °C (Figure 7a), which is consistent with the literature data [54]. To compare the thermal behavior of the ARs, the degradation temperatures corresponding to 10, 30, and 50% weight loss (*T*_10_, *T*_30_, and *T*_50_), and the residues at 550 °C of resins are presented in Table 5. *T_50_* ranged from 380 to 413 °C for different formulations. The thermal stability of these resins was found to be high enough to be used for surface coating applications. The thermal stability of resins follows this order: SFO-G < LSO-G < SFO-P < LSO-P. The increasing number of –OH groups in the alkyd formulation and the formation of hydrogen bonds between –OH groups may have an effect on increasing the thermal stability [55]. Moreover, PE has a stable neopentyl core structure with four reactive primary hydroxyl groups, which can be responsible for providing a high degree of branching and the increased thermal stability of ARs [37]. The derivative thermogram (DTG) curves (Figure 7b) show a multiple-step decomposition of FDCA-based AR samples attributed to the decomposition of the macromolecular chains, followed by the C–O and C–C linkage cleavages [17,55]. Residue at 550 °C ranged from 17.1 to 19.9%. Taking into account that this formulation does not have any mineral filler, the residue must arise as a consequence of a carbonization process in inert conditions, especially from the aromatic groups [56]. SFO-G is characterized by higher *T*_10_ (310 °C), *T*_30_ (350 °C) and *T_max_*_1_ (353 °C) values when compared to PA-SFO-G exhibiting *T*_10_ of 285 °C, *T*_30_ of 338 °C, *T_max_*_1_ of 345 °C (Table 5, Figure 7). Replacing phthalic anhydride with the flat and rigid FDCA furan ring can lead to a decrease in the segmental mobility of the polymer chains. This restriction of internal rotation can increase the thermal decomposition temperature of the resulting polyester compared to PA-based analogs [57].

The Kissinger–Akahira–Sunose (KAS) method was utilized to evaluate the kinetic and thermodynamic characteristics of the synthesized ARs [58,59]. After converting the original TGA data, an analysis was performed to explore the relationship between the conversion *α*, the reaction rate (*dα/dt*), and temperature at different heating rates, as illustrated in Figure S5. The results indicate that in the early stages of pyrolysis, there is a progressive rise in the degree of conversion. As the critical temperature is reached, the conversion rate escalates swiftly before ultimately leveling off at a defined plateau. Furthermore, as the heating rate increases, alkyd samples necessitate a higher temperature to achieve the same decomposition state, leading to an elevated peak temperature. The correlation between *E_a_* and the *α* value, as established through the KAS method, can be categorized into three separate regions, each associated with at least three distinct mechanisms, where each mechanism demonstrates a unique activation energy. The initial region pertains to values of *α* reaching up to 0.3, characterized by a significant rise in *E_a_* related to the volatilization of small molecules or CO_2_ produced from chain ends. The second region (0.3 < *α* < 0.7) exhibits the relatively stable *E*_a_. Finally, the third region (0.7 < *α* < 0.9) exhibits a marked increase in *E*_a_. Increasing the temperature leads to a predominance of random scission in macromolecular chains [55]. The calculated average *E_a_* was found to be 226.6 kJ mol^−1^, aligning with the *E_a_* of alkyds derived from phthalic anhydride and glycerol [55].

**Table 5:** Thermal behavior data of the FDCA-based alkyd coatings.

Alkyd Resin	*T*_10_, °C	*T*_30_, °C	*T*_50_, °C	*T_max_*_1_, °C	*T_max_*_2_, °C	*T_max_*_3_, °C	Residue at 550, %	Reference
PA-SFO-G	285	338	380	345	414	439	8.4	This work
Commercial alkyd resin YF-155	207	311	369				7.49	[60]
Alkyd resin based on recycled PET and hyperbranched polyesters	243	-	388	-	-	-	11.4	[61]
Akd-S coatings	250	310	350	210	345	370	8.1	[62]
Soya alkyd resin	215	305	336	200	340	450	12.0	[56]
SFO-G	310	350	380	353	391	435	19.9	This work
LSO-G	320	357	394	354	392	434	19.8	
SFO-P	320	357	397	365	402	435	17.1	
LSO-P	320	370	413	363	423	-	19.3	
CAR	288	329	376	310	394	437	9.7	

### 3.5. UV Stability of FDCA-Based Alkyd Coatings

UV radiation is recognized as a significant factor contributing to the detrimental effects on polymers and polymer composite materials, as it triggers photochemical reactions that result in irreversible damage to the material [63]. UV light, a part of solar radiation, is an invisible spectrum with wavelengths ranging from 100 to 400 nm. The impact of UV irradiation results in various physical and chemical alterations within polymer matrices. Significant results encompass macromolecular rearrangement, a reduction in molecular weight resulting from chain scission, changes in crystallinity, discoloration, and a decrease in mechanical performance. Unsaturated groups, along with additives like plasticizers, antioxidants, and stabilizers present in plastics, initially absorb UV light. This process leads to the formation of oxygen radicals, which play a role in the degradation of polymer chains. Nonetheless, the specific polymer type and its chemical structure play vital roles in influencing the pathways and outcomes of UV-induced degradation, underscoring the necessity of examining individual polymers [64]. Indoor materials are exposed to sunlight that has passed through window glass, rather than being directly illuminated by the sun. The UVA-351 lamp is a valuable instrument for accelerating testing processes while safeguarding interior materials from excessive strain. It effectively replicates sunlight as it traverses window glass, rendering it suitable for assessments of both indoor and automotive interiors. A UV climate test chamber functions as a specialized device for environmental testing, aimed at assessing the durability of polymers and coatings when subjected to simulated weathering conditions.

The FTIR-ATR analyses proved the changes in FTIR spectra of AR during photo-oxidative aging and degradation (Figure S6). After 24 h of UV irradiation there was broadening of the carbonyl peak (at approximately 1726 cm^−1^), signifying the formation of additional oxygen-bearing species on the surfaces of the films [65]. The C=O stretching vibration assigned to the ester group at 1720 cm^−1^ can broaden and shift to 1700–1695 cm^−1^ due to carboxylic acid formation. Moreover, acid peroxide species are commonly evidenced by the bands at 1780–1790 cm^−1^ as a product of alkyd photodegradation [66]. The surface properties of the analyzed coating products after UV test were assessed by water contact angle measurements [66]. The contact angles of water with the surfaces of the films decreased by ~1.2–1.4 times as the films were irradiated under UV light (Figure S7, Table S1), signifying an increase in surface hydrophilicity caused by the formation of additional chemical species containing oxygen on the film surfaces [65]. These phenomena can be linked to the formation of photo-oxidation aging and degradation species over time, which was confirmed with FTIR. 

After UV irradiation, ARs can undergo degradation, which can lead to changes in their thermal stability [67]. TG/DTG analysis of the coatings after UV demonstrates a decrease in the T_10_ for all the studied resins (Figure S8, Table S3). This indicates an increase in the rate of thermal degradation of the polymer matrix after exposure of the coating to UV radiation, which leads to chain breakage and the formation of free radicals that accelerate thermal decomposition [67]. The amount of residue after thermal decomposition varies depending on the type of resin. The increase in residue after 550 °C is due to the decomposition of organic matter under the influence of UV radiation, which leads to an increase in the mineral content of the coating. However, a more profound study of the photodegradation of FDCA-based alkyd resins is required.

### 3.6. Application of the FDCA-Based ARs for Wood Coating

**Figure 8:**
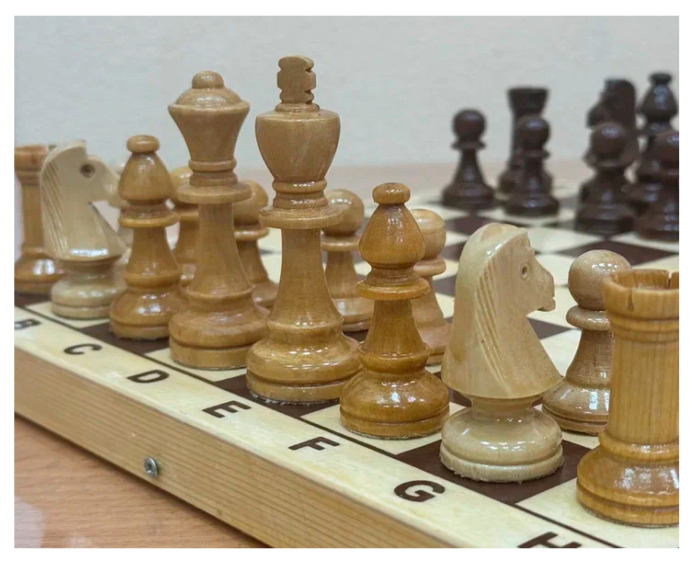
FDCA-based ARs for coating wood chess pieces.

Alkyd varnish functions as a superior finish for wooden chess pieces and boards, offering a durable, protective layer that can attain either a glossy or satin look, while also highlighting the natural beauty of the wood. This application establishes a safeguard against moisture and dirt, enabling the application of multiple layers to attain a denser, more durable finish. Figure 8 illustrates the visual characteristics of alkyd coatings on chess pieces. The coatings demonstrate effective leveling and aesthetic quality, validating the diverse coloration and gloss levels. Additionally, these bio-alkyd coatings successfully maintain the natural appearance of the wood surface.

## 4. Conclusions

Considering the increasing transition from traditional alkyd coatings to bio-based and renewable formulations due to environmental regulations, there is still a notable deficiency in experimental studies regarding the synthesis and functional properties of biorenewable FDCA-based ARs. This study demonstrated the feasibility of using FDCA to produce ARs, which hold promise for coating applications. Various vegetable oils, including sunflower seed and linseed oils, were used along with pentaerythritol and glycerol as polyols. The formation of the ARs was confirmed using FTIR and ^1^H NMR methods. The AV below 15 mg KOH g^−1^ and HV below 70 mg KOH g^−1^ for the synthesized ARs fall within acceptable limits, indicating their suitability for industrial applications. A reduction in tack-free time from 5 to 3 h and an enhancement in pencil hardness from 2H to 3H were observed when HV increased from 29.3 to 69.5 mg KOH g^−1^. The incorporation of pentaerythritol as polyol enhances the viscosity, hardness, and thermal stability of FDCA-based ARs when compared to glycerol. The shear strength and contact angle of linseed oil-modified ARs increased, while the tack-free time decreased compared to sunflower seed oil-modified ARs. This is attributed to higher degrees of unsaturation, which enhance the crosslinking process and/or polymerization per unit weight of material. All synthesized ARs demonstrated exceptional adhesion to metal plates, as evidenced by completely smooth cut edges (classification 0—0% detached coating). The thermal stability of the synthesized ARs up to 220 °C was found to be high enough to be used for surface coating applications. *T*_50_ ranged from 380 to 413 °C for different formulations and achieved the highest value for FDCA-based AR modified with linseed oil using pentaerythritol as a polyol component. The |Z|_0.02 Hz_ values for LSO-G, SFO-P and LSO-P exceed 10^6^ Ω cm^−2^, suggesting effective protection against the erosion of corrosive media to the substrate and demonstrating the high corrosion resistance, comparable to that of CAR. The alkyd coatings applied to chess pieces exhibit excellent leveling and aesthetic quality, validating the diverse coloration and gloss achieved. Furthermore, these bio-alkyd coatings successfully maintain the natural appearance of the wood surface. FDCA-based ARs demonstrated reduced tack-free time, improved thermomechanical properties, and comparable hardness when contrasted with phthalic anhydride-based ARs, highlighting the promise of FDCA as a sustainable substitute for phthalic anhydride in AR formulation, incorporating a higher percentage of renewable materials for wood coating applications.

This study highlights several potential ways for future investigation. The focus areas encompass examining a broader spectrum of vegetable oils with different levels of unsaturation to optimize drying time and crosslink density; studying the effect of UV stabilizers or the hybridization of the synthesized ARs with other biopolymers on their durability for indoor and outdoor use; and performing a life cycle assessment for industrial scalability and environmental advantages of these bio-based ARs in relation to their petroleum-based alternatives.

## Figures and Tables

**Table 1:** Formulations of the FDCA-based ARs.

Ingredients, Parts by Weight	Formulations				
	SFO-G	LSO-G	SFO-P	LSO-P	PA-Based SFO-G
Sunflower seed oil	70.0	-	70.0	-	70.0
Linseed oil	-	70.0	-	70.0	-
Glycerol	8.8	8.8	-	-	8.8
Pentaerythritol	-	-	9.76	9.76	-
FDCA	19.9				-
Phthalic anhydride	-	-	-	-	18.9
Maleic anhydride	1.0				
Na_2_CO_3_	2.8				
Zinc acetate	0.1				
Calcium drier	1.3				
Cobalt drier	1.3				

**Table 2:** Physicochemical properties of the FDCA-based ARs.

Properties	Sample				
	SFO-G	LSO-G	SFO-P	LSO-P	PA-Based SFO-G
AV, mg KOH g^−1^	13.9 ± 0.7	14.3 ± 0.6	13.1 ± 0.5	12.9 ± 0.6	11.8 ± 0.5
HV, mg KOH g^−1^	29.3 ± 1.5	38.8 ± 1.7	59.7 ± 2.4	69.5 ± 2.8	31.4 ± 1.7
Density (at 23.0 ± 0.5 °C), g cm^−3^	0.746 ± 0.03	0.855 ± 0.04	0.758 ± 0.03	0.887 ± 0.05	0.751 ± 0.02
Viscosity, mm^2^ s^−1^	570.4 ± 20.5	51.0 ± 3.0	684.6 ± 15.3	124.2 ± 6.0	493.7 ± 15.7
Color (Gardner scale)	18	13	16	14	16

**Table 3:** Physicochemical properties of the FDCA-based alkyd coatings.

Properties	Sample				
	SFO-G	LSO-G	SFO-P	LSO-P	PA-Based SFO-G
Tack-free time, h	5	4	4	3	6
Lap shear strength, N mm^−2^	91.9 ± 1.5	103.6 ± 1.7	99.6 ± 1.6	110.3 ± 1.9	89.8 ± 2.1
Pencil hardness	2H	2H	3H	3H	2H
Adhesion	0	0	0	0	0
Contact angle, °	51.4 ± 1.4	58.9 ± 1.3	54.8 ± 1.2	63.9 ± 1.4	52.7 ± 1.5

**Table 4:** Chemical resistance properties of the FDCA-based alkyd coatings.

Resin	n-Hexane	n-Heptane	Isopropanol	Acetone	Toluene	Benzene
SFO-G	1	1	1	2	2	2
LSO-G	1	1	1	2	2	2
SFO-P	1	1	1	2	2	2
LSO-P	1	1	1	2	2	2

1—completely unaffected, 2—color changed, film faintly swelled.

## Data Availability

The data that support the findings of this study are available from the corresponding author upon reasonable request.

## Supplementary Materials

The following supporting information can be downloaded at: https://www.mdpi.com/article/10.3390/polym17223022/s1. Figure S1: Setup for the synthesis of ARs; Figure S2: ^1^H NMR spectra of sunflower seed oil (a), glycerides (b), and the SFO-P (c); Figure S3: Digital images of SFO-G (a), LSO-G (b), SFO-P (c), LSO-P (d) coatings after an adhesion test; Figure S4: Digital images of SFO-G (a), LSO-G (b), SFO-P (c), LSO-P (d) coatings after a salt spray chamber (chamber capacity of 240 L, temperature of 40 °C, salt spray fall-out rate of 4.0 mL h^−1^, pH of 7, exposure to salt mist for 3 h and humidity for 1 h); Figure S5: Temperature-dependent conversion α, the reaction rate (dα/dt) and DTG curves at different heating rates; Table S1: Variation in activation energy of the composite resin coating with relative conversion ratio; Figure S6: Comparison of FTIR spectra of SFO-G (a), LSO-G (b), SFO-P (c), LSO-P (d) coatings in the range of 1900–1500 cm^−1^ before and after UV aging; Table S2: Comparison of contact angle values before and after UV aging; Figure S7: Evaluation of contact angles for SFO-G (a), LSO-G (b), SFO-P (c), LSO-P (d) coatings after UV aging; Figure S8: TG/DTG curves of SFO-G (a), LSO-G (b), SFO-P (c), LSO-P (d) coatings after UV aging; Table S3: Thermal behavior data of the FDCA-based AR after UV aging.

## Abbreviations

The following abbreviations are used in this manuscript:

ARs	Alkyd resins
CAR	Commercial alkyd resin
FDCA	2,5-Furandicarboxylic acid
FTIR	Fourier Transform Infrared
NMR	Nuclear Magnetic Resonance
UV	Ultraviolet
AV	Acid value
HV	Hydroxyl value
EIS	Electrochemical impedance spectroscopy
OCP	Open circuit potential
KAS	Kissinger–Akahira–Sunose
TGA	Thermogravimetric Analysis
TG	Thermogravimetry
DTG	Derivative Thermogravimetry
