# Supplementary material for: Biorenewable FDCA-Based Alkyd Resins for More Sustainable Wood Coatings
Source: Polymers (Basel). 2025 Nov 14;17(22):3022. doi: 10.3390/polym17223022 (PMC12655958; doi:10.3390/polym17223022)
Supplement: Supplementary file 1 [file polymers-17-03022-s001.zip › polymers-3963062-supplementary.pdf]

Biorenewable FDCA-based alkyd resins for more sustainable wood coatings

Victor Klushin [1,2], Ivan Zubkov [1,3], Dmitry Petrenko [1], Alina Petrenko [1], Tatyana Yurieva [1], Tatyana Belichenko [1], Aleksey Yatsenko [1], Yash Kataria [1] and Anna Ulyankina [1,*]

[1] Platov South-Russian State Polytechnic University (NPI), Research Institute of Nanotechnology and New Materials, Novocherkassk 346428, Russia;
victorxtf@yandex.ru (V.K.); ivan.n.zubkov@yandex.ru (I.Z.); dmitrypetrenko1998@yandex.ru (D.P.); alinazelens@yandex.ru (A.P.); yurevatanya06@gmail.com (T.Y.); tanya.bond.12@yandex.ru (T.B.);
alexyats-npi@yandex.ru (A.Y.); katariayash1603@gmail.com (Y.K.)
[2] Don State Technical University, Scientific and Educational Center "Materials", Rostov-on-Don 344002, Russia
[3] Don State Technical University, Resource Center for Collective Use of the Scientific and Educational Center "Materials", Rostov-on-Don 344002, Russia

* Correspondence: anya-barbashova@yandex.ru

Content

Figure S1 Setup for the synthesis of ARs 2

Figure S2 1H NMR spectra of sunflower seed oil (a), glycerides (b), and the SFO-P (c) 3

Figure S3 Digital images of SFO-G (a), LSO-G (b), SFO-P (c), LSO-P (d) coatings after an adhesion test 4

Figure S4 Digital images of SFO-G (a), LSO-G (b), SFO-P (c), LSO-P (d) coatings after a salt spray chamber (chamber capacity of 240 L, temperature of 40 °C, salt spray fall-out rate of 4.0 mL h^{-1}, pH of 7, exposure to salt mist for 3 hours and humidity for 1 hour) 4

Figure S5 Temperature-dependent conversion α, the reaction rate ($d\alpha/dt$) and DTG curves at different heating rates 5

Table S1 Variation of activation energy of the composite resin coating with relative conversion ratio 5

Figure S6 Comparison of FTIR spectra of SFO-G (a), LSO-G (b), SFO-P (c), LSO-P (d) coatings in the range of 1900–1500 cm^{-1} before and after UV aging 6

Table S2 Comparison of contact angle values before and after UV aging 6

Figure S7 Evaluation of contact angles for SFO-G (a), LSO-G (b), SFO-P (c), LSO-P (d) coatings after UV aging 6

Figure S8 TG/DTG curves of SFO-G (a), LSO-G (b), SFO-P (c), LSO-P (d) coatings after UV aging 7

Table S3 Thermal behavior data of the FDCA-based AR after UV aging 7

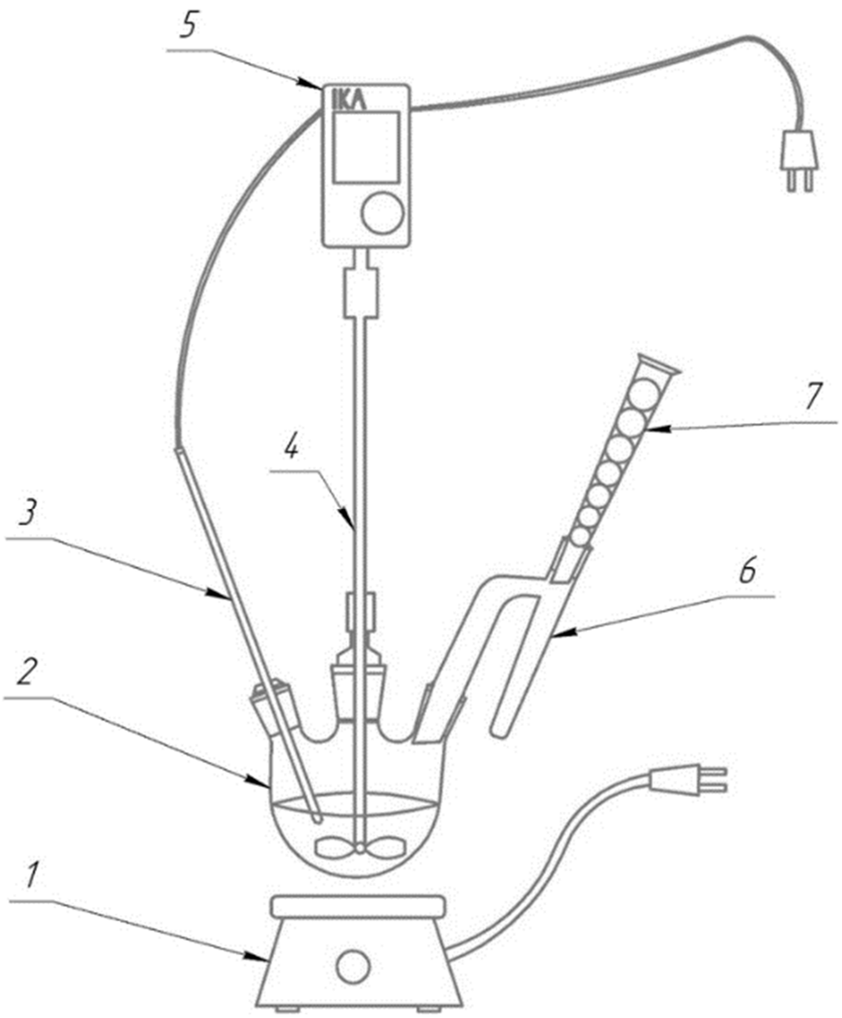

Figure S1 Setup for the synthesis of ARs: 1 – Hotplate; 2 – Reaction flask; 3 – Thermometer; 4 – Stirrer; 5 – Stirrer motor/controller; 6 – Dean-Stark apparatus; 7 – Condenser

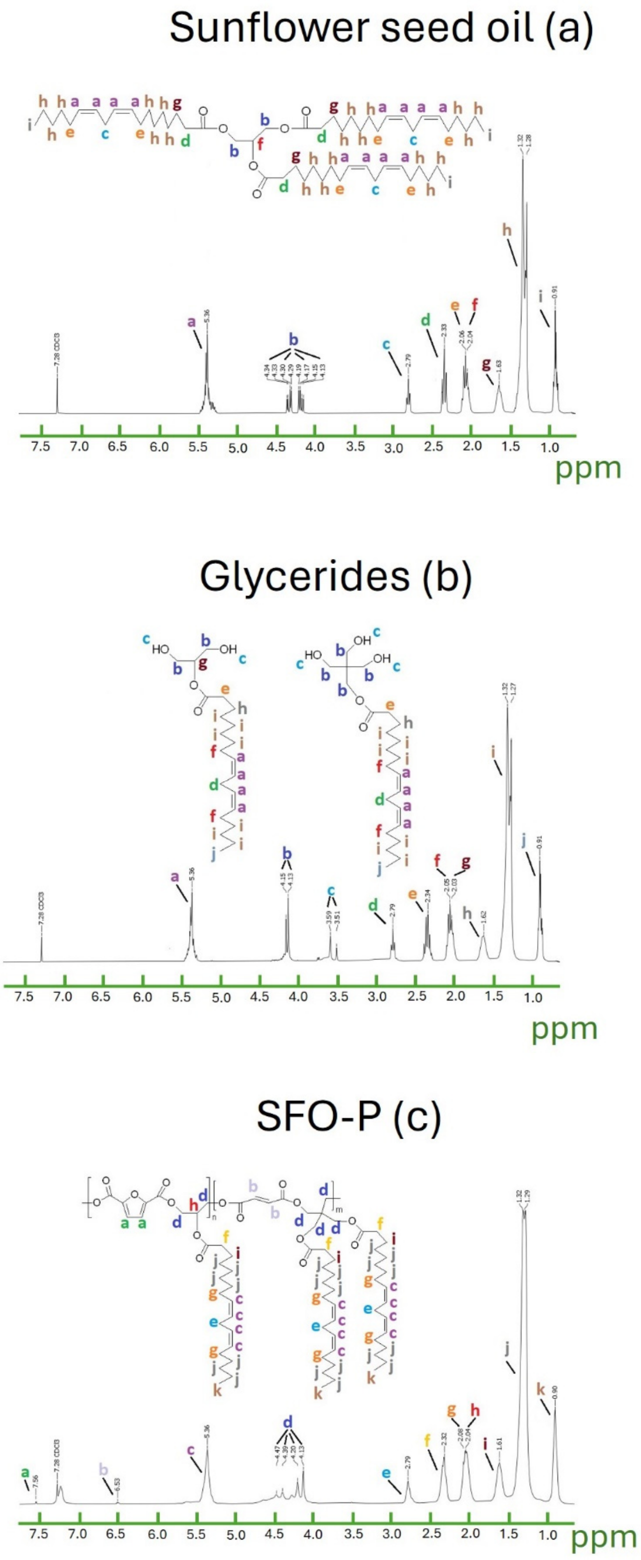

Figure S2 ^{1}H NMR spectra of sunflower seed oil (a), glycerides (b), and the SFO-P (c)

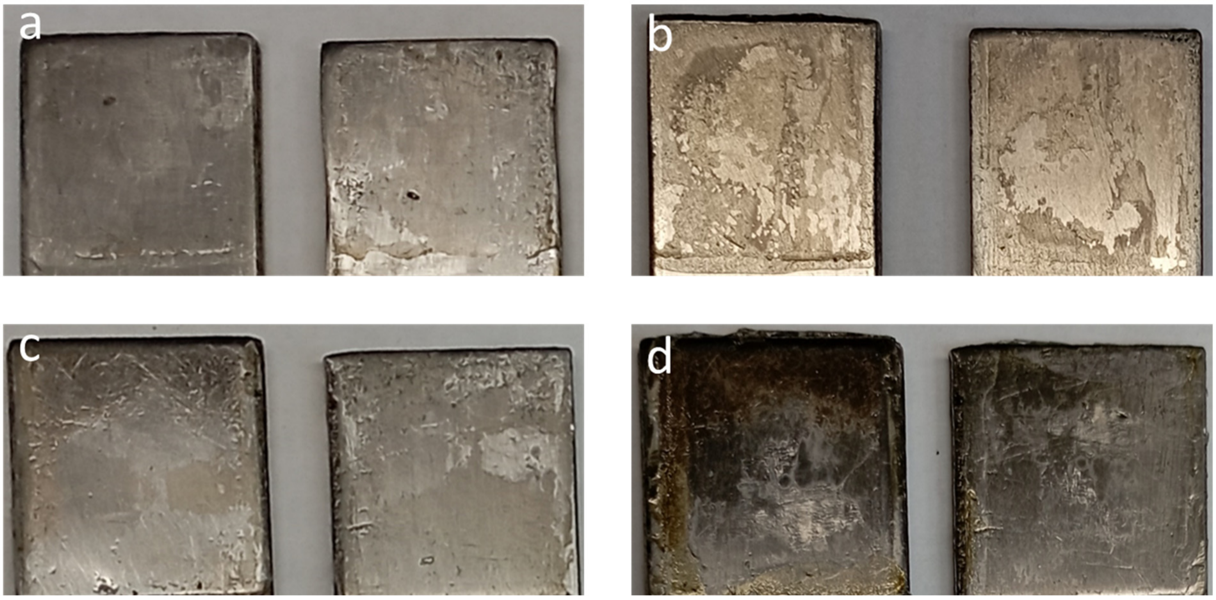

Figure S3 Digital images of SFO-G (a), LSO-G (b), SFO-P (c), LSO-P (d) coatings after an adhesion test

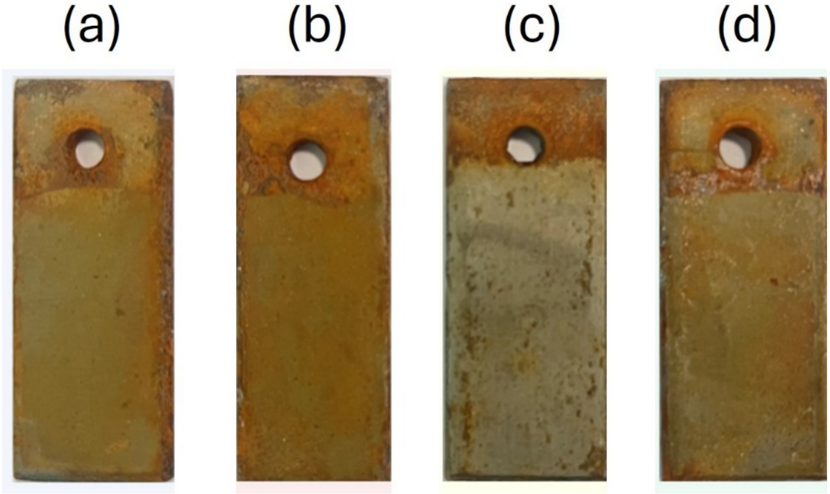

Figure S4 Digital images of SFO-G (a), LSO-G (b), SFO-P (c), LSO-P (d) coatings after a salt spray chamber (chamber capacity of 240 L, temperature of 40 °C, salt spray fall-out rate of 4.0 mL h^{-1}, pH of 7, exposure to salt mist for 3 hours and humidity for 1 hour)

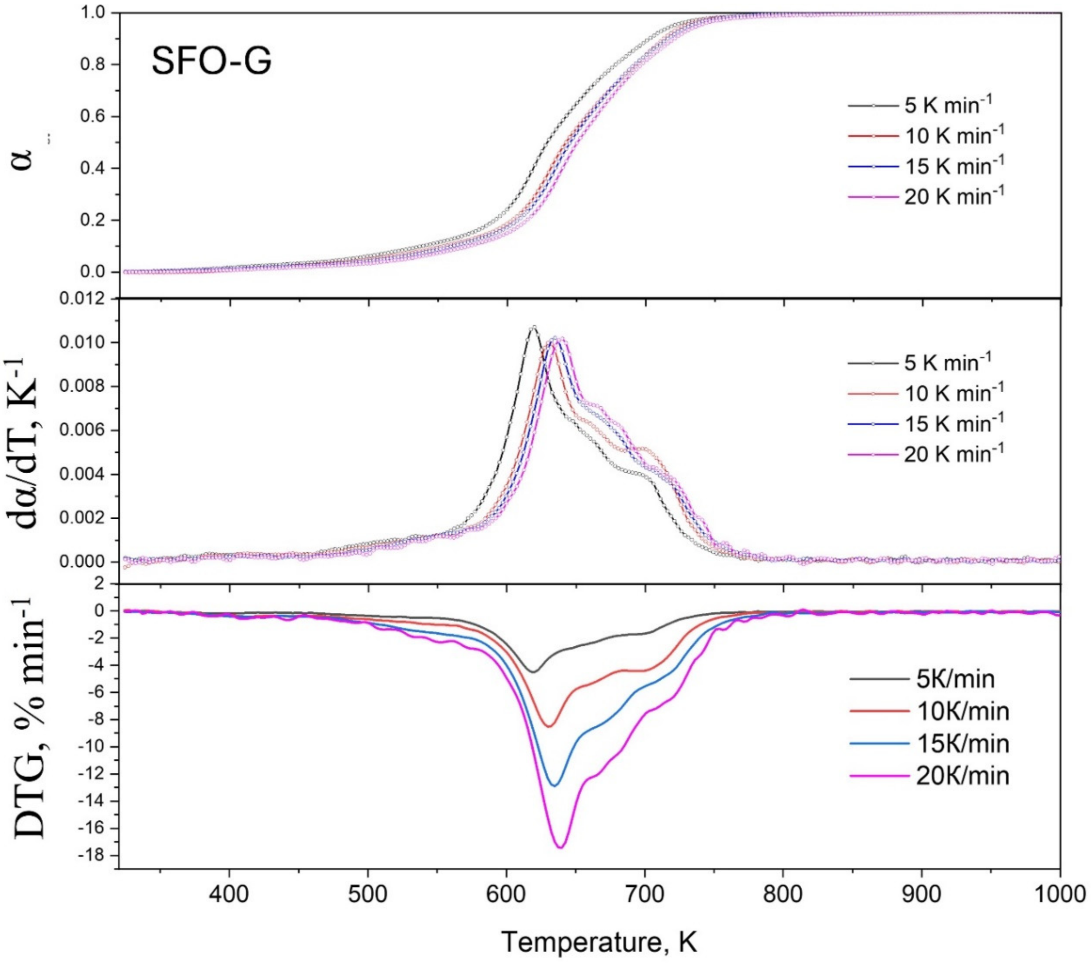

Figure S5 Temperature-dependent conversion α, the reaction rate (*dα/dt*) and DTG curves at different heating rates

Table S1 Variation of activation energy of the composite resin coating with relative conversion ratio

α	Fitted curves	E_a, kJ mol^{-1}	R^2
0.2	y = − 19,435.46× + 21.78	161.58	0.9956
0.3	y = − 24,109.95× + 28.4	200.44	0.9955
0.4	y = − 25,014.80× + 29.14	207.97	0.9870
0.5	y = − 24,914.78× + 28.30	207.14	0.9743
0.6	y = − 26055.71× + 29.22	216.62	0.9568
0.7	y = − 27174.27× + 29.91	225.92	0.9046
0.8	y = − 33682.17× + 38.13	280.03	0.9273
0.9	y = − 37683.74× + 42.14	313.30	0.9774
Average		226.62	

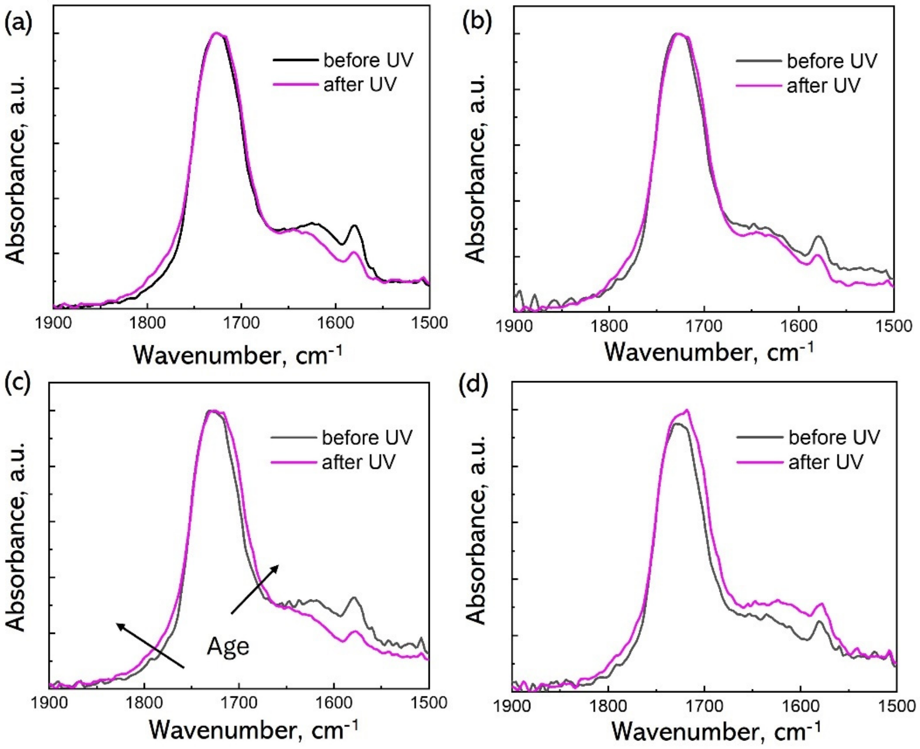

Figure S6 Comparison of FTIR spectra of SFO-G (a), LSO-G (b), SFO-P (c), LSO-P (d) coatings in the range of 1900–1500 cm^{-1} before and after UV aging

Table S2 Comparison of contact angle values before and after UV aging

Contact angle, °	Sample			
	SFO-G	SFO-P	LSO-G	LSO-P
Before UV test	51.4	58.9	54.8	63.9
After UV test	44.7	46.7	44.6	47.4

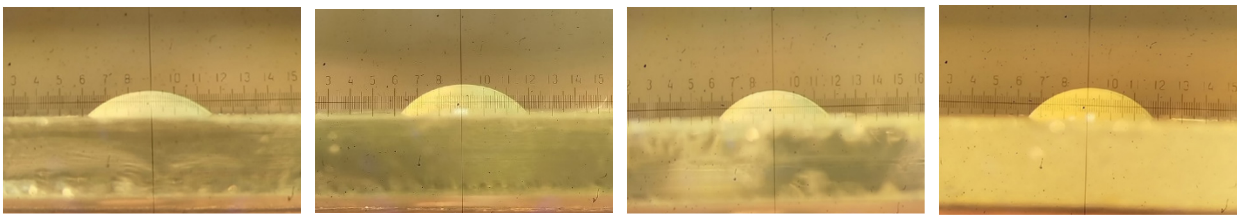

a **b** **c** **d**

Figure S7 Evaluation of contact angles for SFO-G (a), LSO-G (b), SFO-P (c), LSO-P (d) coatings after UV aging

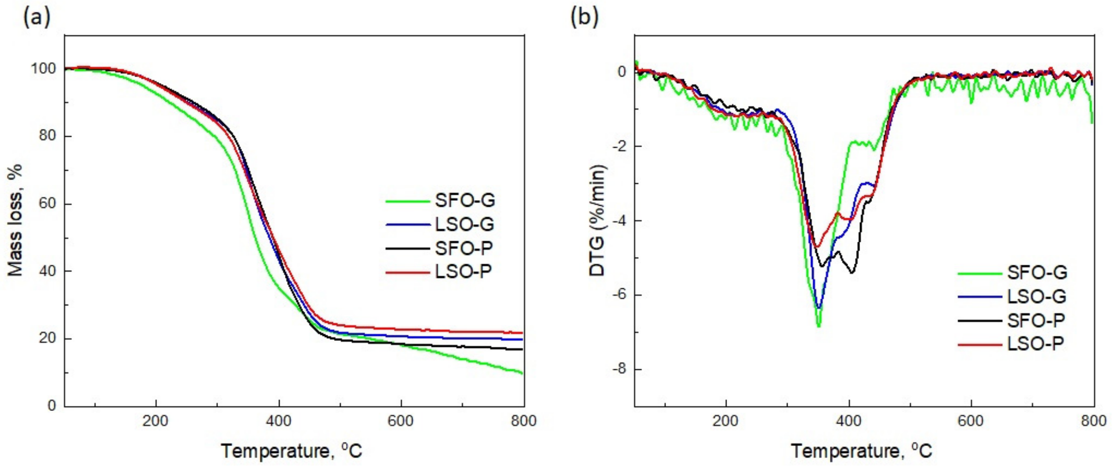

Figure S8 TG/DTG curves of SFO-G (a), LSO-G (b), SFO-P (c), LSO-P (d) coatings after UV aging

Table S3 Thermal behavior data of the FDCA-based AR after UV aging

Alkyd resin	T_{10}, °C	T_{30}, °C	T_{50}, °C	T_{max1}, °C	T_{max2}, °C	T_{max3}, °C	Residue at 550, %
SFO-G	215	328	361	351	-	427	19.9
LSO-G	251	347	384	351	385	440	21.2
SFO-P	261	350	390	356	405	433	19.0
LSO-P	248	344	390	347	404	427	23.3
